# Occlusive retinal vasculopathy on ultrawide-field fluorescein angiography in intraocular cytomegalovirus infection: a case series

**DOI:** 10.1186/s12348-026-00606-w

**Published:** 2026-06-23

**Authors:** Anamika Patel, Ilaria Testi, Harry Petrushkin, Carlos Pavesio

**Affiliations:** Uveitis Department, Moorfields Eye Hospital, 162 City Rd, London, EC1V 2PD UK

## Abstract

**Purpose:**

To describe ultrawide-field fluorescein angiographic features of extensive retinal capillary non-perfusion in eyes with intraocular cytomegalovirus infection.

**Methods:**

Retrospective, single-centre observational study.

**Results:**

Five patients (six eyes) with intraocular CMV infection were included (mean age 62 years; range 20–84). All eyes showed widespread retinal haemorrhages without features of classical oedematous/haemorrhagic necrotising cytomegalovirus retinitis. In eyes with retinitis, the lesions showed a granular phenotype.Three eyes presented with hypertensive uveitis and raised intraocular pressure without neovascularisation. Ultrawide-field fluorescein angiography demonstrated extensive capillary non-perfusion involving both arterioles and venules, reflecting a predominantly occlusive vasculopathy. Vitreous haemorrhage occurred in three eyes, and one progressed to phthisis bulbi despite virological control. Predisposing factors included haematological malignancy, severe malnutrition, local corticosteroid implantation, diabetes with preceding shingles, and systemic immunomodulatory therapy. All the patients received systemic and intravitreal anti viral treatment and were additionally treated for the raised intraocular pressure and respective complications secondary to retinal ischemia.

**Conclusion:**

Intraocular cytomegalovirus infection in non-human-immunodeficiency-virus patients may present as hypertensive uveitis with an occlusive pan-retinal vasculopathy, with or without retinitis, best recognised on ultrawide-field fluorescein angiography. Antiviral therapy and laser may achieve initial control but are insufficient alone; long-term monitoring and patient counselling are essential to address recurrent haemorrhage and neovascular glaucoma.

## Introduction

Cytomegalovirus retinitis occurs across a wide clinical and immunological spectrum, from patients with severe immunosuppression, such as human immunodeficiency virus infection, to non-human-immunodeficiency-virus patients receiving systemic immunosuppression or with more subtle immune dysregulation. It can occur in those receiving systemic immunosuppression for malignancy, autoimmune diseases, or transplantation, but also in individuals with diabetes, age-related immune decline or local steroid exposure [1, 2]. Unlike the minimally inflamed form seen in HIV-related CMV, non-HIV cases often show vitritis, anterior uveitis, and marked, usually arterial, vasculitis with or without small areas of retinitis [1, 2]. Schneider et al. [3] described chronic retinal necrosis as a smouldering CMV variant with widespread retinal occlusive vasculitis and delayed neovascular changes, pointing to the central role of vascular injury and ischaemia in this disease.

Although vascular involvement is increasingly recognised, the true extent and pattern of retinal ischaemia in non-HIV CMV infection remain poorly characterised. Earlier studies have often underrepresented the degree of microvascular compromise, which may exist before or beyond clinically visible retinitis. Ultra-wide-field fundus fluorescein angiography (UW-FFA) allows a broader evaluation of perfusion and can reveal early or subclinical capillary non-perfusion (CNP).

The aim of this study was to characterise extensive occlusive retinal vasculopathy on ultrawide-field fluorescein angiography in eyes with non-human-immunodeficiency-virus intraocular cytomegalovirus infection and to correlate these findings with clinical phenotype, predisposing immune status, treatment, and outcomes.

## Methodology

This was a retrospective observational study patients diagnosed with intraocular cytomegalovirus infection. Ocular imaging, specifically UW-FFA, was reviewed for all patients with CMVR who underwent angiographic evaluation.

A detailed history was obtained in each case, including systemic medications, immunosuppressive therapy, and overall immune status. Demographic data, such as age, sex, and time of diagnosis, were recorded. Clinical presentation, ocular findings, and ancillary investigations were correlated with UW-FFA features to evaluate the pattern and extent of retinal vascular involvement. One previously published case of cytomegalovirus anterior uveitis/endotheliitis with occlusive retinal vasculopathy but no retinitis was also included to illustrate a comparable angiographic phenotype of intraocular cytomegalovirus-associated retinal ischaemia [2].

This retrospective case series was conducted in accordance with institutional requirements and adhered to the tenets of the Declaration of Helsinki. Formal institutional review board approval was not required for this retrospective analysis of anonymised clinical data. Written informed consent for publication of clinical details and images was obtained where required.

## Results: (Table 1)

**Table 1:** Clinical characteristics, complications, and outcomes in intraocular cytomegalovirus-associated occlusive vasculopathy

Case	Age / sex	Underlying condition / predisposing factor	IOP at presentation → final	NVI during follow-up	Visual acuity at presentation → final	Complications	Follow-up period (months)
1	20 / M	Acute lymphoblastic leukaemia; post–bone marrow transplant	53 → 11 mmHg	Yes	Counting fingers → no light perception / phthisis bulbi	Hyphaema, tractional retinal detachment, fibrovascular proliferation, vitreous haemorrhage	88
2	79 / M	Hypertension; chronic obstructive pulmonary disease; severe malnutrition/frailty	26 → 16 mmHg	Yes	Hand movements → hand movements	Neovascularisation of the iris; vitreous haemorrhage	13
3	68 / M	Biopsy-proven sarcoidosis; prior intravitreal fluocinolone acetonide implant	38 → 20 mmHg	No	6/60 → 6/48	Nil	28
4	62 / M	Diabetes/metabolic dysregulation; hypertension; asthma; preceding herpes zoster	32 → 8 mmHg	No	6/18 → 6/24	Vitreous haemorrhage; stable after vitrectomy and endolaser photocoagulation	34
5	61 / M	Systemic corticosteroids and mycophenolate mofetil	10 → 10 mmHg BE	No	RE 6/24, LE 3/60 → RE 6/6, LE 6/7.5	Nil	48

M-Male, NVI- Neovascularisation of Iris, BE – both eyes, RE- Right eye, LE- left eye

### Case 1

A 15-year-old boy with a history of acute lymphoblastic leukaemia (ALL) diagnosed in 2013, with relapse in 2018, underwent allogeneic bone-marrow transplant in July 2018. Following treatment for CMV viraemia with intravenous foscarnet and discontinuation of maintenance therapy with oral valganciclovir, as well as cessation of immunosuppressive therapy (ciclosporin) two weeks earlier, the patient presented to the emergency department with sudden, painless visual loss in the right eye.

**Fig. 1:**
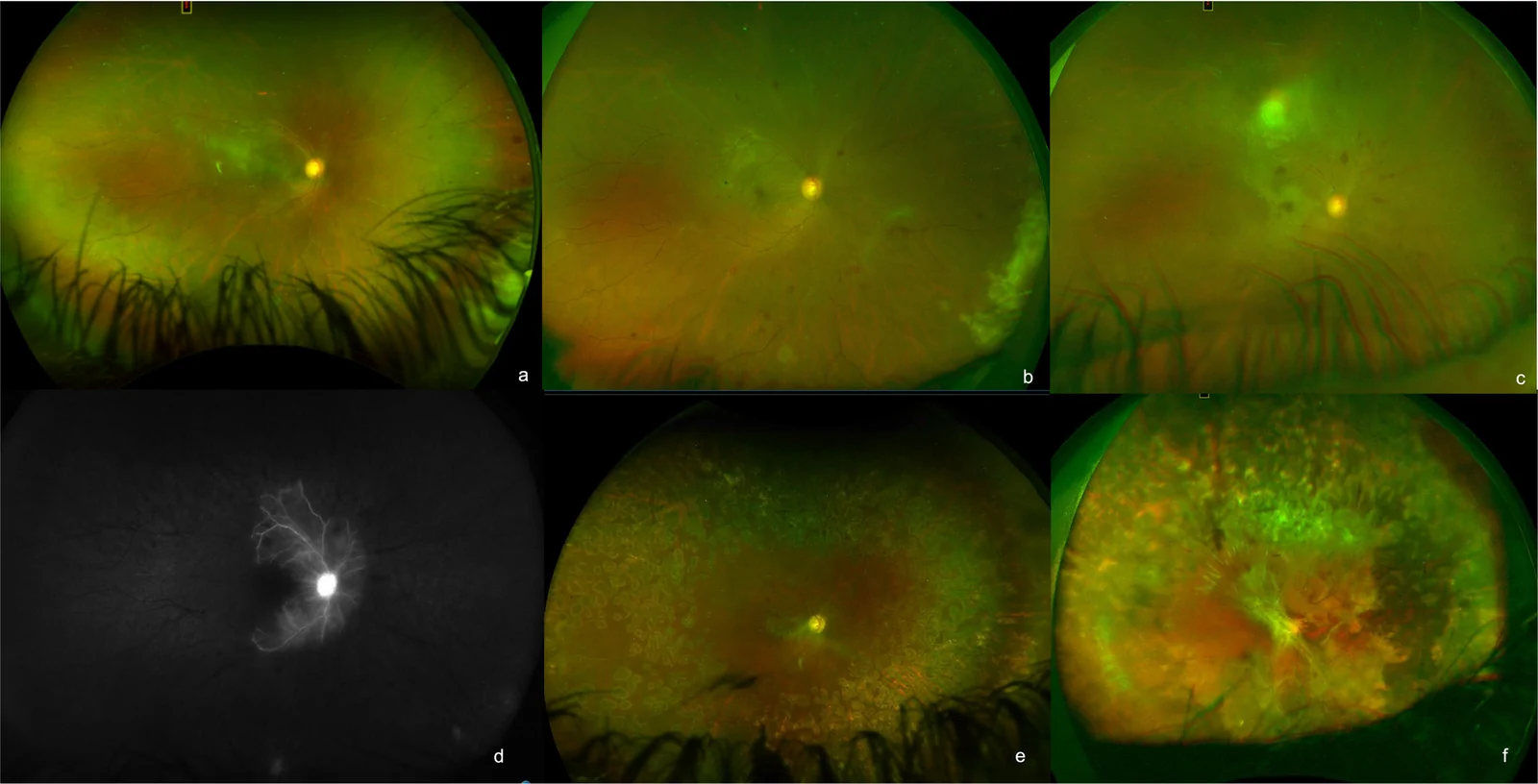
Sequential colour fundus and wide-field fluorescein angiography (FFA) images of Case 1 showing the evolution of occlusive cytomegalovirus (CMV) retinitis. (**a**) day 0 (presentation): granular retinitis with scattered intraretinal haemorrhages and markedly elevated intraocular pressure, consistent with hypertensive uveitis. (**b**) day 7: progression of retinal haemorrhages with increased vascular sheathing and segmental arteriolar narrowing. (**c**) day 28: deep intraretinal haemorrhages and macular retinal whitening. (**d**) day 28 ffa: extensive capillary non-perfusion involving both arterioles and venules. (**e**) eight weeks post-presentation: post-laser (panretinal photocoagulation) image showing resolution of active retinitis with residual vessel sclerosis and optic disc pallor. (**f**) nine months from presentation: development of vitreous hemorrhage and fibrovascular proliferation leading to localised traction

On examination, the right eye demonstrated counting-finger vision and a markedly elevated intraocular pressure of 53 mmHg, consistent with hypertensive uveitis. Fundus evaluation revealed peripheral retinitis with segmental vascular interruption resembling Kyrieleis-type arteriolitis. (Fig. 1) While awaiting confirmation of the aqueous humour tap results for CMV, a presumptive diagnosis of CMVR was made. Treatment was initiated with oral valganciclovir, intravitreal foscarnet injections, and both topical and systemic intraocular pressure (IOP)–lowering agents. The diagnosis was subsequently confirmed following laboratory results.

By day 7, vascular changes had intensified, with greater vessel attenuation and intraretinal haemorrhages. As IOP normalised, new macular whitening suggested an evolving arterial occlusion. UW-FFA confirmed widespread capillary non-perfusion extending from the periphery to the posterior pole, underscoring the extensive vascular compromise. The patient underwent panretinal photocoagulation under general anaesthesia.

Six months after presentation, the fundus remained stable with laser marks, but by nine months, spontaneous hyphaema and fibrovascular proliferation appeared, leading to a localised tractional retinal detachment. Visual acuity declined to hand-movements, and despite management, the eye progressed to phthisis bulbi. The left eye remained unaffected throughout follow-up.

### Case 2

A 79-year-old man of Iranian descent with a history of hypertension, chronic obstructive pulmonary disease (COPD), and unintentional weight loss of approximately 20 kg over eight months reported progressive frailty, poor appetite, and reduced ability to prepare meals, contributing to ongoing malnutrition.

He presented with sudden blurring of vision in the right eye. There was no rubeosis iridis, and intraocular pressure measured 26 mmHg. The left examination was unremarkable. Visual acuity in the right eye was hand-movements. Fundus examination revealed granular mid-peripheral retinitis with peripheral haemorrhages and attenuated, sclerosed posterior retinal vessels. The aqueous humour tap for CMV was performed, and intravitreal foscarnet was administered empirically.

**Fig. 2:**
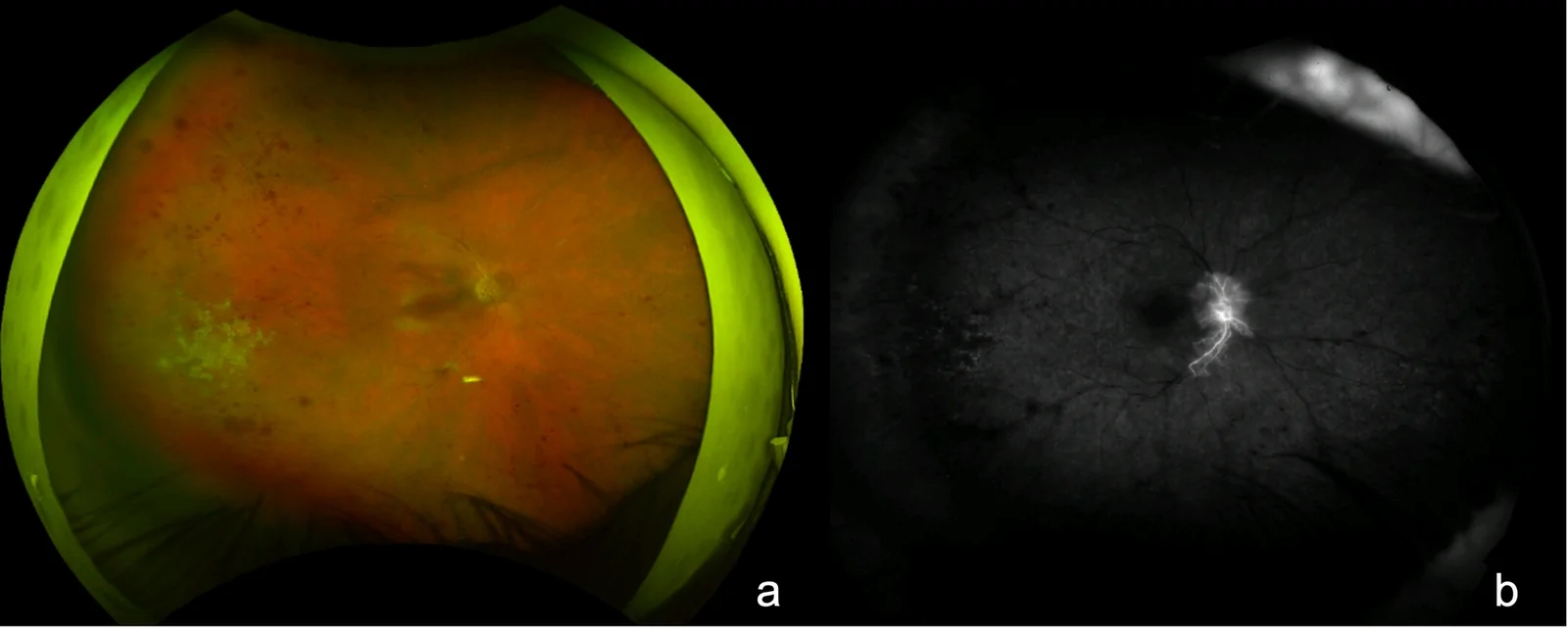
(**a**–**b**) colour fundus and corresponding wide-field fluorescein angiography (FFA) images of case 2. (**a**) colour fundus photograph showing granular retinitis at the mid-peripheral retina with diffuse dot–blot haemorrhages and sclerosis of the retinal vasculature, resulting in minimal perfusion. (**b**) corresponding FFA image demonstrating extensive capillary non-perfusion with only faint residual perfusion of the optic nerve head

The following day, UW-FFA demonstrated extensive capillary non-perfusion with near-total arterial and venous occlusion, sparing only a few perfused vessels at the optic disc. (Fig. 2) PCR analysis of the aqueous sample was positive for CMV, while HSV and VZV were negative. The patient was commenced on oral valganciclovir. Systemic investigations for HIV, syphilis, and primary immunodeficiency were negative.

Four weeks later, he developed a vitreous haemorrhage and secondary rubeosis iridis. Intravitreal bevacizumab was administered, followed by pars plana vitrectomy with endolaser photocoagulation for persistent non-clearing haemorrhage. The BCVA remained HM despite treatment and IOP remained stable.

### Case 3

An 68-year-old man with biopsy-proven pulmonary sarcoidosis and a history of bilateral intermediate uveitis presented to the emergency department with blurred vision in the left eye. He had previously received an intravitreal fluocinolone acetonide (Iluvien) implant 14 months earlier for cystoid macular oedema and was not on systemic immunosuppression at presentation.

Visual acuity was 6/60 in the left eye, and intraocular pressure measured 38 mmHg. Slit-lamp examination revealed fine keratic precipitates on the endothelium. The patient was commenced on tapering topical corticosteroids and dorzolamide with timolol twice daily and referred for review in the uveitis clinic three weeks later.

**Fig. 3:**
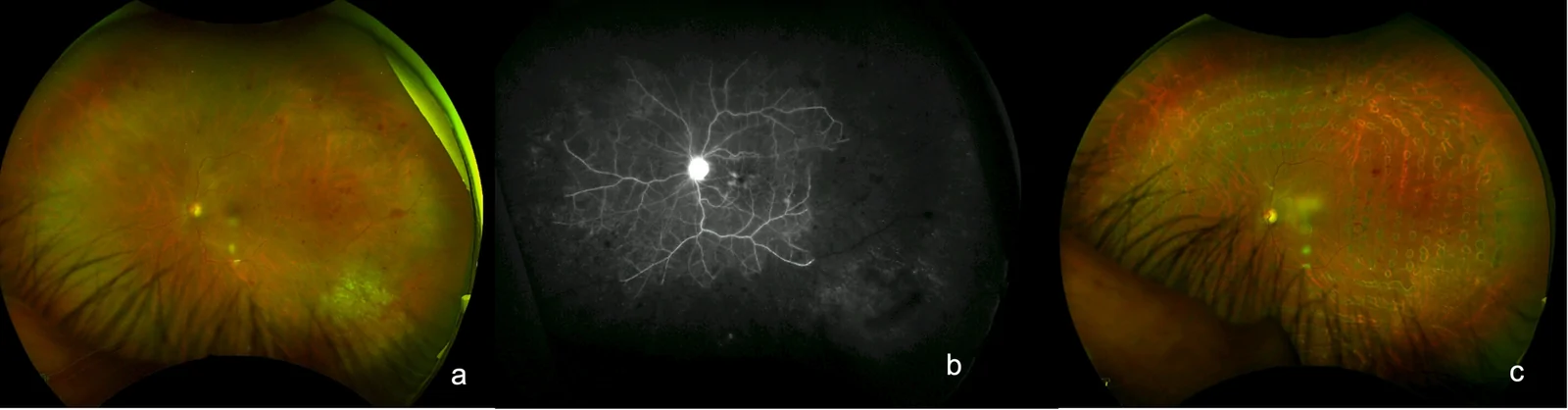
(**a**–**c**) sequential colour fundus and wide-field fluorescein angiography (FFA) images of case 3. (**a**) colour fundus photograph showing granular retinitis in the inferotemporal quadrant with multiple dot-and-blot haemorrhages and sclerosed inferotemporal retinal vessels. (**b**) corresponding FFA demonstrating a hot optic disc, capillary leakage at the macula, and almost 360-degree retinal capillary non-perfusion secondary to combined occlusion of both retinal veins and arterioles. (**c**) post-resolution fundus image showing healed retinitis with well-defined laser scars following panretinal photocoagulation

At follow-up, fundus examination of the left eye showed multiple dot and blot intraretinal haemorrhages admixed with Roth spots, without venous dilation or tortuosity. Granular retinitis was noted in the inferotemporal quadrant, associated with arteriolar inflammation and intermittent vascular sclerosis involving both arteries and veins. UW-FFA revealed areas of blocked hypofluorescence corresponding to the retinitis and extensive peripheral capillary non-perfusion extending circumferentially, with greatest prominence temporally. Additionally, a petaloid pattern of hyperfluorescence was observed at the macula, consistent with cystoid macular oedema (Fig. 3).

The anterior chamber tap tested positive for CMV DNA, confirming the diagnosis of CMV retinitis. Oral valganciclovir therapy was initiated. However, due to deranged renal function and the need for ongoing intravitreal foscarnet therapy, the intravitreal Iluvien implant was removed, as local corticosteroid-induced immunosuppression was considered a predisposing factor for infection. Panretinal photocoagulation was subsequently performed. The BCVA at the last follow-up of was 6/48 and IOP remained stable at 20mmHg.

### Case 4

A 62-year-old man of Caucasian descent with a history of diabetes mellitus, hypertension, and asthma presented with reduced vision in the right eye. He had experienced right-sided herpes zoster involving the torso three months prior to presentation.

**Fig. 4:**
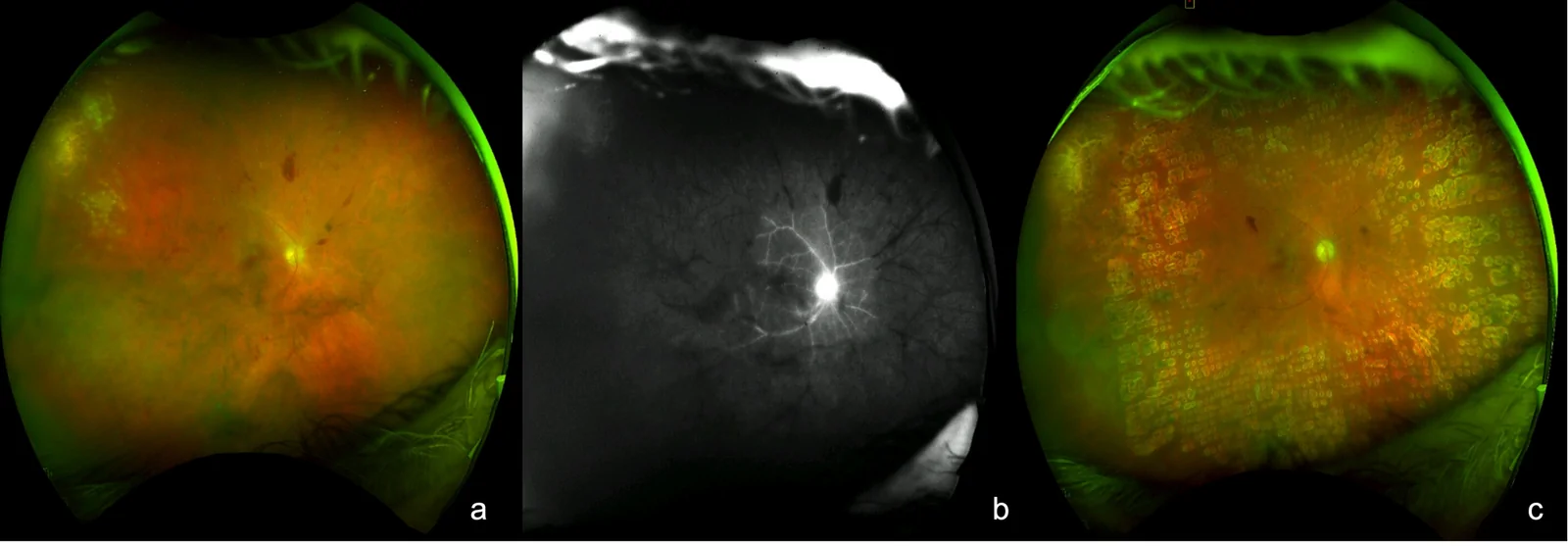
(**a**–**c**) sequential colour fundus and wide-field fluorescein angiography (FFA) images of case 4. (**a**) colour fundus photograph showing granular retinitis in the superotemporal quadrant with diffuse vitritis and deep intraretinal haemorrhages. (**b**) corresponding FFA demonstrating extensive capillary non-perfusion with combined occlusion of both retinal veins and arterioles. (**c**) post-treatment fundus image showing resolution of granular retinitis and vitritis, with laser scars visible following panretinal photocoagulation

At presentation, visual acuity was 6/18 in the right eye and 6/6 in the left, with an intraocular pressure of 32 mmHg in the right eye. Slit-lamp examination showed fine keratic precipitates in the right eye. Fundus evaluation of the right eye revealed grade 2 vitritis and granular retinitis in the nasal quadrant, with intraretinal haemorrhages superiorly. Both arterioles and venules appeared sclerosed and partially occluded, though assessment was limited by vitritis (Fig. 4).

UW-FFA demonstrated widespread capillary non-perfusion, with only a few posterior pole vessels remaining perfused (Fig. 4). An anterior-chamber tap was performed, and the patient was started on oral valganciclovir 900 mg twice daily, intravitreal foscarnet, and topical and systemic IOP-lowering therapy.

Panretinal photocoagulation was undertaken once media clarity permitted. The eye remained stable for several months before developing a vitreous haemorrhage, requiring vitreoretinal surgery with endolaser photocoagulation. At the most recent follow-up, visual acuity had improved to 6/24 in the right eye following vitrectomy and laser treatment with IOP under control of 10mmHg.

### Case 5 [2]

A 61-year-old man of South-East Asian descent developed bilateral cytomegalovirus (CMV) endotheliitis while receiving systemic corticosteroids and mycophenolate mofetil for anterior and intermediate uveitis. The ocular findings were confined to the anterior segment, characterised by corneal oedema with Descemet’s membrane folds and distinctive coin-shaped keratic precipitates, in the absence of retinitis or choroidal involvement.

Polymerase-chain-reaction analysis of the aqueous humour confirmed CMV DNA positivity. The patient was treated with topical ganciclovir 0.15% eye gel five times daily and oral valganciclovir 900 mg twice daily, with continuation of topical and oral corticosteroids. Over subsequent weeks, the endotheliitis resolved almost completely, though (UW-FFA) demonstrated progressive peripheral capillary non-perfusion without active retinal lesions, necessitating panretinal photocoagulation.

## Discussion

In this series, ultra-wide-field fundus fluorescein angiography showed that intraocular CMV infection in non-HIV patients can present as a predominantly occlusive vasculopathy rather than a classic necrotising retinitis. All patients with retinitis demonstrated the granular variant of CMV retinitis, usually confined to a single quadrant and accompanied by deep intraretinal and dot–blot haemorrhages. In several eyes, areas of capillary non-perfusion extended well beyond the visible retinitis and involved both arterioles and venules, suggesting a diffuse microvascular process driven by CMV’s endothelial affinity and a dysregulated immune response [3-5].

Vascular involvement was pronounced. Segmental, beaded arteriolar narrowing and sclerosis were seen in four eyes, affecting both arteries and veins, and two eyes showed macular arterial occlusion at presentation. Even the previously published case without retinitis exhibited peripheral dot–blot haemorrhages and progressive peripheral ischaemia, reinforcing that CMV-related vascular injury can occur independently of overt retinal necrosis [2].

A distinct feature across our cohort was the presentation with hypertensive uveitis—markedly raised intraocular pressure at onset, without neovascularisation. This differs from secondary pressure elevation seen in neovascular glaucoma and likely reflects early trabecular inflammation triggered by viral activity. Recognising this presentation is important, as it may precede visible retinitis or widespread vascular occlusion [5] (Table 1).

The spectrum of predisposing factors was broad. Case 2 suggests that severe malnutrition and frailty may act as permissive factors for cytomegalovirus reactivation in late life. Case 4 illustrates a possible contribution from metabolic or immune dysregulation in the setting of diabetes and recent herpes zoster infection. The previously reported bilateral case further shows that intraocular cytomegalovirus may produce progressive retinal ischaemia even when disease is confined clinically to the anterior segment.

The systemic and local backgrounds post–bone marrow transplantation, malnutrition, local corticosteroid implant, sarcoidosis, diabetes with preceding shingles, and systemic immunomodulatory therapy illustrate the breadth of immune imbalance in which CMV reactivation can occur. Most eyes presented with widespread haemorrhages and vascular sclerosis rather than fulminant haemorrhagic necrotising retinitis typical of HIV-related disease. Vitreous haemorrhage developed in three eyes and iris neovascularisation in two, even after virological control, indicating that established vascular injury rather than viral replication determines long-term outcome.

Our findings are consistent with the arteritic tendency and chronic ischaemic evolution described by Pathanapitoon et al. [3] and Schneider et al. [4] but add further insight into the frequency of hypertensive uveitis and the degree of panretinal capillary non-perfusion seen on angiography. Although panretinal photocoagulation remains essential, it is often insufficient on its own to prevent late sequelae. Persistent ischaemia may continue to drive recurrent vitreous haemorrhage or neovascular glaucoma despite antiviral suppression [6]. Continuous follow-up and clear patient counselling about recurrence and the chronic nature of the disease are critical for preserving vision.

In summary, non-HIV intraocular CMV infection behaves as an occlusive microangiopathy that can evolve independently of necrotising retinitis. Early Ultra-wide-field fundus fluorescein angiography helps reveal the true extent of ischaemia, while awareness of hypertensive uveitis as an early presentation may aid timely recognition and improve outcomes in this under-recognised clinical entity.

## Conclusion

Intraocular cytomegalovirus infection may present as a predominantly occlusive retinal vasculopathy, with non-perfusion extending beyond clinically apparent retinitis and involving both arterioles and venules. Ultrawide-field fluorescein angiography is valuable for defining the full ischaemic burden. Although antiviral therapy and panretinal photocoagulation may achieve initial disease control, these eyes remain at risk of recurrent vitreous haemorrhage and neovascular glaucoma. Long-term vascular surveillance and patient counselling are therefore essential, as further intervention may be required to preserve vision.

## Data Availability

All data and images pertaining to the cases are available with the corresponding author.
